# Diseases of the Blood and Blood-Vessels

**Published:** 1905-02-18

**Authors:** 


### DISEASES OF THE BLOOD AND BLOOD-VESSELS.

(*Continued from page* 317.)

Splenic Anæmia.—Cases of splenic anæmia have been recorded by Stevens[5] and by Springthorpe.[6] The disease is characterised by splenic enlargement associated with more or less pallor, the blood shows the chlorotic type of anæmia with an absence of leucocytosis, and often the presence of leukopenia. It is usually a chronic affection; hæmatemesis often occurs, and sometimes ascites is present. The recognition of the disease is important, as there is reason to believe that many cases are curable by splenectomy. Stevens says that in diagnosis the other causes of splenic enlargement associated with anæmia must first be excluded. These are rickets, syphilis, malaria, albuminoid disease, tuberculosis, leukæmia, Hodgkin's disease, hepatic cirrhosis, and even, in rare cases, pernicious anæmia. The term Banti's disease has been applied to that variety of splenic anæmia in which it is associated with hepatic cirrhosis and ascites. The six cases of splenic anæmia recorded by Springthorpe are remarkable in that they all occurred in one family, and that splenectomy was performed on two of the cases. The series consisted of three sisters and one brother, with his son and niece (the last the daughter of a healthy brother). The first case, a woman aged 28 years, gave a history of palpitation and chlorotic tinge for four years, and dyspnœa for three years. She had had epistaxis twice, several feverish attacks. The spleen was down to the umbilicus, the liver was slightly enlarged, and there were hæmic bruits. In spite of iron, bone marrow, adrenalin, arsenic, and hæmatogen she grew slowly worse. The white cells were unchanged, the red cells fell to 2,350,000 per cubic mm. and the hæmoglobin to 70 per cent. Splenectomy was then done with the result that the red cells rose in numbers to 4,480,000 and the hæmoglobin to 85 per cent. She continued free from pain and from the symptoms of severe anæmia. The excised spleen, which weighed 43 oz. after being in spirit, had a thickened capsule and a great increase in the fibrillar framework of the spleen pulp, made up of the anastomosing processes of endothelial cells, with their rounded or elongated nuclei. The Malpighian bodies were very few, both in absolute numbers and in the number of cells they contained; they gave the impression of having been encroached upon by the fibrosis of the surrounding pulp. Another sister, aged 19, also operated upon with success, had much the same symptoms and physical signs, and the spleen presented the same features. A third sister, aged 23, was similarly affected, also a brother, aged 30 years. The latter had a son, aged 7 years, with similar features, and a niece, aged 10, who had a slightly enlarged spleen, 2,564,000 red cells and 55 per cent. hæmoglobin. The causation in these cases was quite unknown, the patients became affected at various ages, and had suffered from the disease for very various periods before coming under observation. The one patent fact was that both patients were much better in every way without their spleens than with them, though they are not in a state of rude health. The possible indications for splenectomy are (1) malarial spleen; (2) splenic anæmia; (3) that variety known as Banti's disease; (4) wandering spleen; (5) abscess of the spleen. It is curious that splenectomy in splenic leukæmia is almost invariably followed by a fatal result. Almost identical pathological changes in the spleen were found in a fatal case published by Strickland, Hodgson, and Anderton.[7]

[5] Brit. Med. Jour., Oct. 8. [6] Lancet. Oct. 8. [7] Ibid, Oct. 1.

